# Role of exercise in cardiovascular health: a narrative review from prevention to therapeutic utilizations

**DOI:** 10.3389/fcvm.2026.1746819

**Published:** 2026-04-22

**Authors:** Yong Wang, Xingbin Du, Qifei Wang

**Affiliations:** Shandong Huayu University of Technology, Dezhou, Shandong, China

**Keywords:** cardiovascular disease, exercise, IGF-1/PI3K/Akt pathway, inflammation, prevention

## Abstract

Cardiovascular disease (CVD) continues to be the leading cause of morbidity and mortality globally, imposing a substantial burden on healthcare systems worldwide. Physical inactivity is a significant modifiable risk factor that contributes to the onset and progression of CVD. Current guidelines recommend regular aerobic and muscle-strengthening exercise, with even below-guideline volumes reducing mortality risk significantly. Notably, even physical activity levels below these recommendations can significantly reduce mortality risk, emphasizing the importance of any movement over a sedentary lifestyle. Exercise functions as both a preventive and therapeutic intervention, helping individuals with and without CVD, including those recovering from myocardial infarction or managing heart failure. At the molecular level, the IGF-1/PI3K/Akt signaling pathway plays a crucial role in exercise-induced cardiac protection by promoting balanced cardiac growth, enhancing contractility, and reducing fibrosis. Furthermore, increased endothelial nitric oxide synthase (eNOS) activity improves vascular function, antioxidant enzymes mitigate oxidative stress, and peroxisome proliferator-activated receptor gamma coactivator 1-alpha (PGC-1*α*) stimulates mitochondrial biogenesis, while pro-inflammatory cytokines such as interleukin-6 (IL-6) and tumor necrosis factor-alpha (TNF-α) are downregulated. Large-scale cohort studies have proved that regular exercise can reduce all-cause and CVD mortality by 36%–56%. This magnitude of risk reduction rivals or exceeds that achieved by pharmacological interventions such as statins or antihypertensives, positioning physical activity as a foundational, cost-effective intervention for population-level cardiovascular disease prevention. However, excessive exercise may pose risks such as arrhythmias or myocardial strain, underscoring the need for personalized, balanced exercise programs. Future research should focus on defining best exercise prescriptions, understanding exercise–drug interactions, and developing biomarkers to check adaptive responses. Ultimately, integrating personalized exercise medicine into healthcare and public policy offers a cost-effective strategy for preventing and managing CVD, promoting lifelong cardiovascular resilience and well-being.

## GRAPHICAL ABSTRACT

**Figure:**
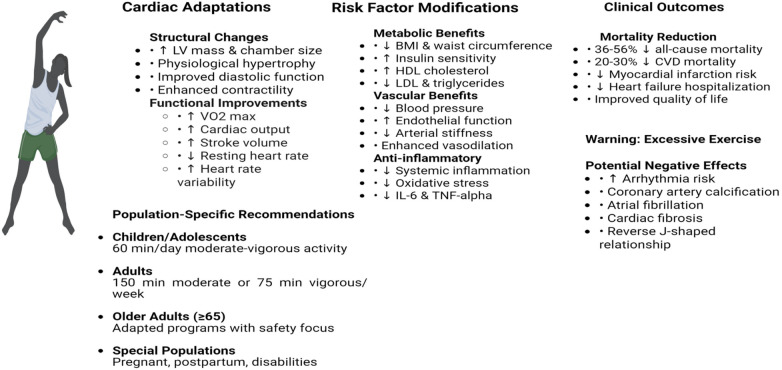
Summary of exercise impacts on cardiovascular health. Exercise induces advantageous cardiac adaptations (structural and functional improvements), risk factor modifications (metabolic, vascular, and anti-inflammatory benefits), and significant clinical outcomes (36%–56% mortality reduction). Population-specific suggestions scope from 60 min/day for youth to 150 min/week moderate or 75 min/week vigorous activity for adults, with adapted programs for older adults and special populations. Excessive exercise may pose risks, comprising arrhythmia and coronary calcification.

## 1 Introduction

Cardiovascular disease (CVD) remains a major cause of illness and death globally, imposing a heavy strain on healthcare systems and communities (1). Recently, in this context, the significance of exercise and physical activity in improving cardiovascular health has obtained significant scientific and clinical attention (2). Wide experimental and clinical literature has consistently reported that participating in regular physical activity is vital in decreasing various CVD risk factors, including high blood pressure, abnormal lipid levels, obesity, insulin resistance, and systemic inflammation (3). These positive impacts promote cardiopulmonary fitness and improve vascular function, autonomic control, and metabolic health, collectively leading to a notable decrease in cardiovascular disease and mortality rates (4).

Importantly, the physiological effects of an acute bout of exercise differ from the adaptations induced by chronic exercise training. A single exercise bout transiently increases heart rate, stroke volume, and cardiac output, and elevates vascular shear stress that acutely modulates endothelial function. With repeated bouts over weeks to months, exercise training produces sustained central adaptations (e.g., increased maximal stroke volume and cardiac output through cardiac remodeling and expanded blood volume) and improves cardiorespiratory fitness (VO₂max/VO₂peak), together enhancing oxygen delivery during exertion. Cardiorespiratory fitness is a robust, independent predictor of cardiovascular events and all-cause mortality in population studies, emphasizing its clinical relevance in both prevention and therapy. Exercise training can also improve coronary endothelial function and flow reserve in patients with established coronary artery disease (CAD), highlighting that vascular adaptations can occur early during training (5–11).

Identifying these advantages, the 2018 Physical Activity Guidelines for Americans provide evidence-based suggestions to facilitate cardiovascular health throughout life. Children and adolescents should engage in at least 60 min of moderate-to-vigorous physical activity daily to encourage cardiovascular development and overall health. For adults, the guidelines recommend taking part in at least 150 min of moderate-intensity aerobic activities, such as brisk walking or cycling, or 75 min of vigorous-intensity aerobic exercises, such as running or high-intensity interval training, each week (12). Moreover, muscle-strengthening exercises involving major muscle groups should be done on two or more days per week to facilitate musculoskeletal health and metabolic function (13). While the cardioprotective benefits of physical activity are profound, it is vital in personalizing exercise plans through individual risk factors, fitness levels, and existing health conditions to enhance advantages and decrease possible risks, such as exercise-driven cardiac events in at-risk groups (14). Based on acknowledging the dose-response correlation between exercise intensity, duration, and frequency, healthcare professionals and public health practitioners can develop targeted interventions (15).

Beyond the general population, the 2019 UK Chief Medical Officer (CMO) physical activity guidelines stress the significance of physical activity for pregnant and postpartum women, older adults (aged ≥65 years), and individuals with disabilities (16). Consistent with the 2020 World Health Organization (WHO) guidelines, all age groups are supported to take part in moderate to vigorous physical activity (17). The prevalence of sedentary lifestyles today implies that numerous people do not obtain the necessary levels of physical activity for the best health (18). However, emerging evidence indicates that even moderate-intensity physical activity confers meaningful health benefits—particularly among previously inactive individuals. Notably, substantial risk reductions are observed even at activity levels below current public health guideline targets, with the greatest relative risk reduction occurring when individuals transition from complete inactivity to any level of regular physical activity (19). Notably, individuals at high CVD risk who engage in low-intensity physical activity exhibit lower mortality rates and greater life expectancy compared with their inactive counterparts (20).

Exercise training has been shown to reduce cardiovascular mortality by approximately 20%–30% in secondary prevention populations and to decrease the incidence of major adverse cardiovascular events (MACE) by around 25% compared to standard medical care alone (21, 22). In primary prevention, dose–response analyses reveal that increasing physical activity from zero to 7.5 metabolic equivalent task (MET)-hours per week yields the greatest reduction in CAD risk, approximately 14%, with progressively smaller but still significant benefits at higher activity levels (23). Supervised exercise interventions have consistently improved cardiorespiratory fitness in patients with CAD, increasing peak oxygen uptake (VO₂ peak) by 11%–36% and reducing systolic blood pressure by 5–7 mmHg (24). In heart failure patients with reduced ejection fraction, the landmark HF-ACTION trial (22) demonstrated that structured aerobic training led to modest but significant reductions in all-cause mortality and hospitalizations (4%–11%). Additionally, the EGEA (Étude du Groupe d'Étude de l'Athérosclérose) observational cohort found that participation in cardiac rehabilitation was associated with dose-dependent improvements in quality-of-life scores, as measured by the Minnesota Living with Heart Failure Questionnaire (MLHFQ) (25). Exercise training is vital in both the prevention and control of heart failure. Animal models that replicate exercise training provide critical insights into the functional and molecular mechanisms that explain the protective benefits of exercise (26, 27). A study utilizing animal evidence on CVD has stressed the benefits of regular exercise training, revealing the fundamental mechanisms behind cardiovascular protection (28).

Evidence indicates that exercise training can reduce cardiovascular risk factors, lower the incidence of cardiovascular events, and enhance antioxidant capacity, collectively contributing to improved cardiac function (29). Moreover, exercise training is related to physical changes, such as cardiac hypertrophy, vascular remodeling, and advantageous modifications in mitochondrial metabolism (30). Preclinical and translational studies further suggest that exercise training induces favorable structural and metabolic remodeling, including physiological hypertrophy, improved mitochondrial function, enhanced antioxidant capacity, and reduced pro-inflammatory signaling, providing mechanistic support for observed clinical benefits (29–33).

This review explores the benefits of exercise training for CVD and how it encourages cardiac protection via different pathways and mechanisms. This insight will help in determining new therapeutic targets and approaches for CVD control. The review compiles recent scientific literature evidence on the intricate correlation between exercise and cardiovascular health, centering on the advantages, possible mechanisms, and essential considerations for effectively incorporating physical activity into CVD prevention and control approaches.

## 2 The correlation between exercise and cardiovascular health

Exercise is a type of physical activity that is intentionally designed, organized, and repeated with the specific goal of improving and maintaining physical fitness (34). In broad terms, physical activity encompasses any bodily movement generated by skeletal muscles that leads to energy expenditure, encompassing a range of domains from activities of daily living and occupational tasks to recreational activities and competitive sports (35). Physical fitness refers to health- or skill-related attributes that result from regular activity. Physical activity is a core factor in improving cardiovascular health, as it significantly impacts different physiological systems that help decrease the risk of CVD (36). Consistent exercise improves cardiopulmonary fitness by enhancing the function of the heart, lungs, and vascular system, thereby promoting oxygen delivery and utilization throughout the body. Beyond hemodynamic improvements, exercise training exerts significant anti-inflammatory effects that contribute to long-term cardiovascular protection. Regular physical activity has been shown to reduce systemic levels of C-reactive protein (CRP), interleukin-6 (IL-6), and tumor necrosis factor-alpha (TNF-α), while increasing anti-inflammatory mediators (e.g., IL-10 and adiponectin) (37). Crucially, studies on pulmonary rehabilitation show that regular physical activity can lower inflammation and increase exercise capacity in people with chronic illnesses and related cardiovascular conditions (38).Mechanistically, the anti-inflammatory effects of regular exercise are thought to be mediated by reductions in visceral adiposity and the repeated induction of an anti-inflammatory cytokine milieu with each exercise bout (9) Improved cardiovascular performance is also evident in lower resting heart rate, increased stroke volume, and enhanced endothelial function, as measured by flow-mediated dilation (FMD) and nitric oxide bioavailability (39).

In addition, exercise training favorably remodels the lipid profile: it increases high-density lipoprotein cholesterol (HDL-C) by 2–8 mg/dL and elevates apolipoprotein A-I (ApoA-I) levels, while enhancing HDL functionality—including cholesterol efflux capacity and paraoxonase-1 (PON1) activity (40). Endurance training also reduces plasma triglycerides by 10%–20% and shifts the LDL particle size distribution away from atherogenic small, dense LDL particles (41). These adaptations are associated with increased lipoprotein lipase (LPL) activity in skeletal muscle and enhanced hepatic LDL receptor expression following chronic physical activity (42). The suggestions from the 2018 Physical Activity Guidelines for Americans are inclusive of all demographics, comprising pregnant and postpartum women, older adults, and individuals with disabilities, stressing the significance of physical activity in various biological and social contexts (43).

Recent explorations have stressed the enhancing benefits of incorporating a variety of exercise forms, such as high-intensity interval training (HIIT), resistance workouts, and mind-body practices, such as yoga and tai chi, in the improvement of cardiovascular health (44). These exercise modalities can promote cardiorespiratory fitness and vascular function, while also improving metabolic health and psychological well-being. Moreover, taking part in regular physical activity is vital in decreasing chronic inflammation and oxidative stress, both of which are linked to the progression of cardiovascular diseases (45). Hence, an individualized method that includes aerobic, strength, and flexibility exercises is proposed to obtain the best cardiovascular advantages, while also considering individual preferences, capabilities, and health conditions. These holistic approaches are critical for promoting adherence and determining long-term enhancements in cardiovascular health (46).

## 3 The correlation between sports and cardiovascular health

While sports are generally categorized as physical activities, they are often characterized by their competitive objectives and skills unique to each sport (46). They can integrate aerobic and anaerobic energy systems, impose unique biomechanical demands, and carry distinct injury and performance considerations. In study and practice, sport-specific activity is sometimes assessed separately from general exercise to capture differential impacts on cardiovascular and metabolic outcomes. Participation in sports is increasingly determined as a powerful means to facilitate cardiovascular health via different physiological modifications (47). Recent literature has stressed that taking part in sports causes exercise-driven cardiac remodeling (EICR), which is defined according to an enhancement in left ventricular mass, enlargement of cavity dimensions, and promoted diastolic function. These structural modifications cause better cardiac output and myocardial effectiveness, hence decreasing the heart's workload during rest (48). In addition, sports commonly involve intermittent high-intensity activities, such as those found in soccer, basketball, and combat sports, which elevate maximal oxygen consumption (VO₂ max), a well-established marker of cardiorespiratory fitness and a predictor of cardiovascular mortality. Sports participation can also effectively impact the core cardiovascular risk factors according to decreasing systemic blood pressure, improving lipid metabolism, and enhancing insulin sensitivity (49). The molecular processes responsible for these advantages include heightened activity of endothelial nitric oxide synthase (eNOS), which can promote endothelial function and vasodilation, coupled with a decrease in systemic inflammation and oxidative stress (50).

Additionally, sports that incorporate dynamic aerobic and resistance components cause advantageous adaptations in autonomic nervous system modulation, causing promoted heart rate variability and a lower resting heart rate. While the cardiovascular benefits of sports are noteworthy, current consensus stresses the significance of individualized cardiac evaluations, notably for competitive athletes, to differentiate between physiological adaptations and pathological conditions (40). Pre-participation screening with electrocardiography and echocardiographic imaging is proposed to lower the risk of sudden cardiac events. Emerging guidelines advocate for a nuanced, athlete-focused method to exercise participation that considers genetic and acquired cardiovascular diseases (51).

## 4 Positive impacts of sports and exercise training on cardiovascular disease

Taking part regularly in sports and organizing physical activities can be vital in cardiovascular health, according to promoting different physiological and molecular factors that reduce the risk of CVD. Exercise regimens cause a decrease in traditional CVD risk factors, such as body mass index (BMI), blood pressure, and total cholesterol (52).

A meta-analysis of randomized controlled trials has reported that consistent moderate activities, such as walking, effectively lower BMI, systolic and diastolic blood pressure, and fasting glucose levels in adults (45). Moreover, engaging adolescents in moderate-to-vigorous physical activities decreases their risk of cardiovascular problems, according to promoting metabolic markers, comprising glucose and insulin levels and waist circumference. Physiologically, exercise can trigger an immediate enhancement in sympathetic nerve activity and catecholamine release, which elevates heart rate and cardiac contractility (53). This response improves cardiac output in the context of enhancing stroke volume and left ventricular end-diastolic volume, hence promoting the delivery of oxygen to the myocardium. Long-term endurance training induces physiological hypertrophy characterized by proportional increases in left ventricular (LV) wall thickness, typically 6–10 mm in athletes vs. 6–9 mm in healthy controls, and LV end-diastolic diameter (55–63 mm), resulting in elevated LV mass (150–300 g) without impairing diastolic function or causing myocardial fibrosis (54). Echocardiographic and cardiac magnetic resonance imaging (MRI) studies have demonstrated that these adaptations are distinct from pathological hypertrophy: exercise-induced remodeling is associated with preserved or enhanced diastolic filling (E/A ratio >1.0), absence of myocardial fibrosis on late gadolinium enhancement (LGE) imaging, and regression of structural changes following detraining (55). These findings support the concept of an “athlete's heart” as a benign, characterized by eccentric left ventricular remodeling with preserved or enhanced diastolic function, reversible adaptation to chronic endurance exercise.

Exercise also facilitates vascular health by promoting endothelial function via increased nitric oxide (NO) generation, supports vasodilation, and avoiding atherogenesis. These advantages expand to the cellular level, where physical activity causes the expression of antioxidant enzymes, such as superoxide dismutase (SOD1 and SOD2), glutathione peroxidase, and catalase, hence decreasing reactive oxygen species (ROS)-driven oxidative stress in the myocardium (56). This antioxidative response protects the heart from ischemia-reperfusion (I/R) injury, reduces infarct size, and promotes recovery after cardiac events. For example, endurance training, such as long-distance running and swimming, has been reported to convert pathological hypertrophic signals into physiological ones, activating protective molecular pathways, such as the insulin growth factor-1/phosphoinositide-3 kinase/protein kinase B (IGF-1/PI3K/Akt) pathway (57).

Various sports can offer unique cardiovascular challenges that cause adaptations in the heart and health advantages for athletes (58). Endurance sports, such as long-distance running and cycling, elicit robust central and peripheral cardiovascular adaptations. Various sports offer distinct cardiovascular stimuli that shape specific adaptations. In randomized training studies, 8 weeks of high-aerobic-intensity interval training improves VO₂ max and is accompanied by an increase in stroke volume, indicating that there have been positive structural or functional changes in the heart (59) When exercise volume is controlled, higher-intensity aerobic training generally yields larger gains in VO₂ max than moderate-intensity training (60). Beyond central adaptations, endurance training promotes peripheral remodeling, including increased mitochondrial content and capillary density in skeletal muscle, thereby improving oxygen extraction and utilization during exercise (61). Intermittent team sports (e.g., soccer, basketball) engage both aerobic and anaerobic metabolism and are associated with improvements in autonomic modulation (e.g., increased heart-rate variability) (62) Swimming provides a low-impact whole-body endurance stimulus and can improve blood pressure and lipid profiles while minimizing joint loading; sport-specific patterns of adaptation likely reflect differences in intensity distribution, muscle recruitment, and total training volume (63).

Moreover, literature has revealed that taking part in sports with dynamic and multidimensional movement patterns may increase vascular function and metabolic flexibility more than single-type exercises. Determining these differences enables clinicians to suggest individualized physical activity plans according to individual health requirements and preferences to perfect cardiovascular protection (64).

## 5 Negative impacts of high-intensity exercise on cardiovascular disease

Engaging in physical exercise offers advantages, yet intense workouts, despite their many benefits, may pose cardiovascular dangers, requiring meticulous attention, especially for those with existing heart conditions. High-intensity exercise is defined as activities that exceed 70%–85% of an individual's maximal oxygen (VO₂ max) uptake and are rated above 7 on a 0–10 scale of perceived exertion (RPE) (65). This type of exercise involves short bursts of intense effort, commonly interspersed with rest or lower-intensity periods, as uncovered in HIIT or competitive endurance sports, such as marathon running and cycling. While high-intensity exercise can promote cardiovascular fitness and metabolism, studies have indicated underlying negative impacts, such as an increased risk of arrhythmia, myocardial infarction, and coronary artery calcification (CAC) (66, 67).

While physiological cardiac remodeling induced by exercise training is generally adaptive, extremely high volumes of intense endurance training—such as >60–90 min per day at >80% VO₂ max sustained over many years—may, in rare cases, lead to maladaptive changes including myocardial fibrosis, atrial dilation, and increased arrhythmic risk, particularly in genetically susceptible individuals (55). However, these extreme outcomes only occur with very high exercise doses. Standard training regimens consistently produce beneficial heart adaptations without any signs of damage (68). This distinction is supported by the reversibility of exercise-induced changes following detraining and the absence of myocardial fibrosis on late gadolinium enhancement (LGE) cardiac MRI (69). Human studies, such as the Measuring Athletes' Risk of Cardiovascular Events (MARC) study, has demonstrated that 16% of middle-aged endurance athletes had CAC scores over one hundred Agatston Units, a marker related to increased coronary risk (70, 71). Importantly, the clinical significance of elevated CAC in athletes remains uncertain; unlike sedentary individuals where CAC predicts events, athletes with high CAC may have different plaque morphology (calcified, stable) and continued exercise does not appear to increase acute coronary event risk in this population (72).

Moreover, the prevalence of atrial fibrillation is higher among those who take part in prolonged high-intensity endurance activities, because of exercise-driven cardiac fibrosis, notably in the atrium and right ventricle (73, 74). However, this risk appears confined to the extreme upper tail of the exercise dose–response curve (>5 h/week for >10 years) and must be weighed against the 30%–50% reduction in all-cause mortality associated with regular vigorous exercise (75). Clinically, this suggests that screening for atrial remodeling may be warranted in ultra-endurance athletes but should not discourage prescription of vigorous exercise to standard-risk patients (76).

Fortunately, the fibrotic alterations noted can be reversed via rest and the utilization of medications that inhibit angiotensin II receptors, such as losartan (77, 78). In athletes engaging in high-intensity sports, there is commonly an elevation in biomarkers, such as procollagen type I carboxy-terminal pro-peptide and tissue inhibitors of metalloproteinases, which signal ongoing tissue remodeling (79). Hence, analyzing these biomarkers in conjunction with cardiac morphological and functional assessments provides useful insights into an individual's exercise tolerance and cardiovascular risk. Sports differ in their intensity and cardiovascular needs; for instance, marathon running and competitive cycling are high-intensity endurance activities, while team sports, such as soccer and basketball involve intermittent high-intensity efforts combined with moderate aerobic conditioning (80). Resistance training or strength sports mainly cause cardiac stress via pressure overload, highlighting the requirement for individualized training programs according to individual risk profiles (81).

Overall, while high-intensity exercise improves fitness and cardiovascular adaptations, there is a reverse J-shaped correlation, where excessive volume and intensity can elevate cardiovascular risk. individualized risk evaluation and exercise planning that balances intensity, duration, and rest are critical to enhancing advantages and minimizing the risks linked to intense physical activity (82).

## 6 Mechanistic insights into exercise-driven cardiovascular benefits

### 6.1 IGF-1/PI3K/Akt signaling in exercise training

Exercise training induces specific temporal activation of the IGF-1/PI3K/Akt cascade that differs qualitatively from acute exercise bouts. In rodent models, 4–6 weeks of treadmill training increases cardiac IGF-1 protein expression by 1.8–2.5-fold and enhances IGF-1 receptor autophosphorylation at tyrosine 1131. This sustained ligand-receptor interaction recruits the p85 regulatory subunit of class IA PI3K to the sarcolemma, facilitating PIP2-to-PIP3 conversion with subsequent Akt activation. Notably, chronic training results in sustained Akt phosphorylation at Ser473 (2.3-fold increase) and Thr308 (1.9-fold increase) (83). This persistent activation distinguishes exercise-induced physiological growth from pathological hypertrophy, as the latter typically involves transient Akt activation followed by calcineurin-NFAT signaling (84). The sustained PI3K/Akt activity explains why exercise-trained hearts demonstrate superior functional reserve and resistance to ischemic injury compared to sedentary or pathologically loaded hearts (85).

**Figure 1:**
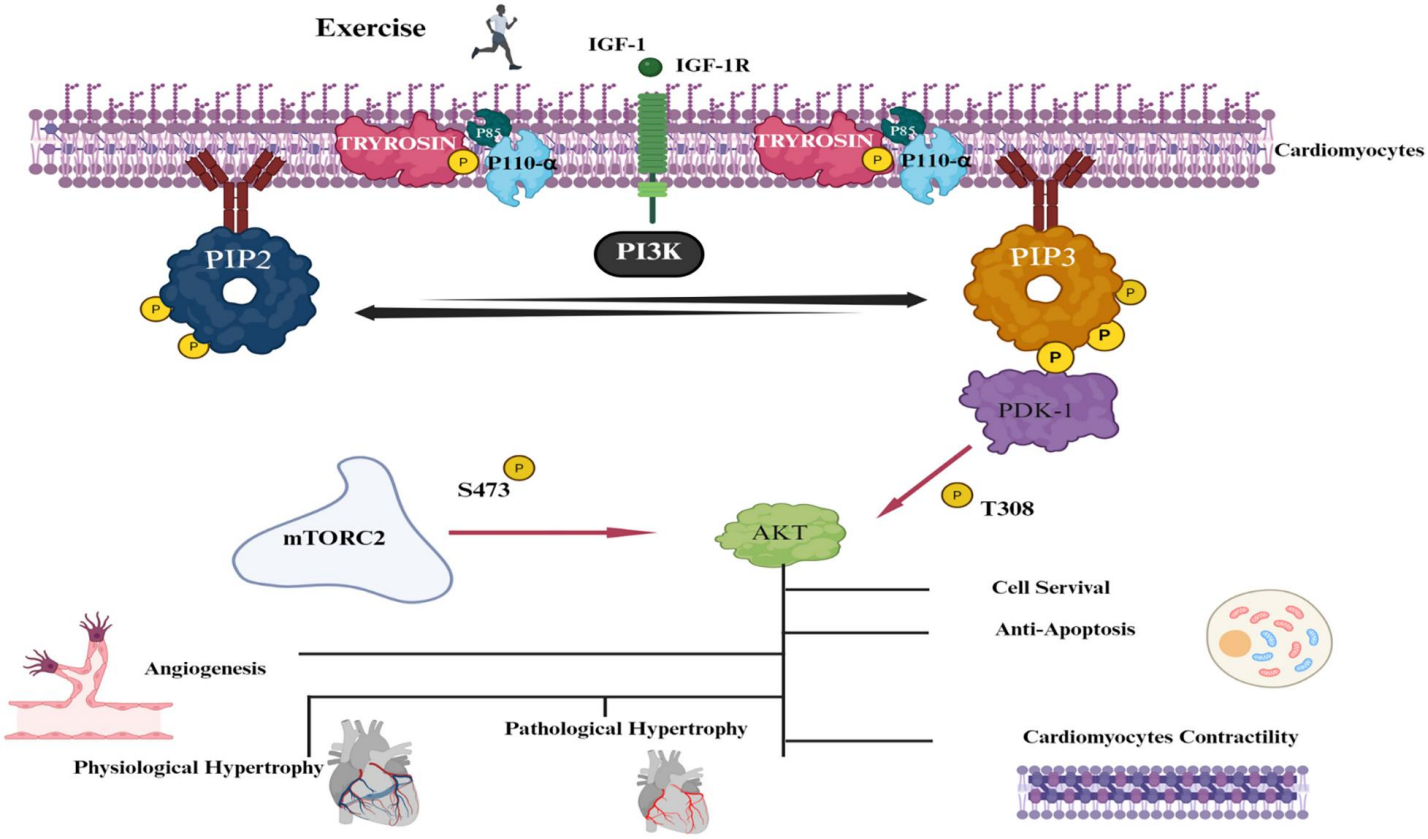
Exercise-driven IGF-1/PI3K/Akt signaling cascade and downstream cardioprotective. Physical activity stimulates the release of IGF-1, which activates IGF-1R and attracts PI3K. This enzyme eases the conversion of PIP2 to PIP3 via phosphorylation. PIP3 then draws Akt and PDK1 to the cell membrane, where Akt is phosphorylated at two sites: T308 according to PDK1 and S473 according to mTORC2. Once activated, Akt facilitates several protective impacts on the heart, such as improving cell survival, avoiding apoptosis, promoting angiogenesis, encouraging physiological cardiac hypertrophy (growth without fibrosis), and promoting the contractility of cardiomyocytes. These impacts differentiate the advantageous remodeling from exercise from pathological hypertrophy.

Fitts et al. (2024) demonstrate that these adaptations are dose-dependent: training volumes >150 min/week moderate intensity are required to sustain PI3K/Akt-mediated physiological hypertrophy, with lower volumes failing to produce significant structural remodeling (86). The downstream effects include: (1) phosphorylation of Bad (Ser136) and FoxO3a (Thr32), reducing cardiomyocyte apoptosis by 40%–60% in ischemia-reperfusion models; (2) activation of mTORC1 (measured by p70S6K phosphorylation) promoting protein synthesis without fibrotic collagen deposition; and (3) inhibition of glycogen synthase kinase-3β (GSK-3β), preventing pathological hypertrophic remodeling. This pathway specifically mediates training-induced eccentric hypertrophy—increasing left ventricular end-diastolic volume by 15%–20% while maintaining wall thickness-to-radius ratios <0.42, distinguishing it from concentric pathological hypertrophy (86) (Figure 1).

### 6.2 Mitochondria-associated membranes and training-induced calcium handling

An emerging mechanism distinct from acute exercise involves exercise training-induced remodeling of mitochondria-associated membranes (MAM)—tethering domains between the sarcoplasmic reticulum (SR) and mitochondria that regulate calcium flux and apoptotic signaling. Chronic endurance training increases the protein density of MAM tethering complexes, specifically the IP3R2-Grp75-VDAC1 interaction, by 35%–50% in cardiac tissue (87). This structural adaptation enhances localized calcium transfer from SR to mitochondria during systole, increasing ATP synthesis efficiency by approximately 25% while preventing cytosolic calcium overload and subsequent calcineurin activation (88).

Training also modulates MAM-resident proteins governing mitochondrial dynamics. Eight weeks of swimming training in rodent models downregulates dynamin-related protein 1 (Drp1) phosphorylation at Ser616 (a fission-promoting modification) by 40%, while increasing mitofusin-2 (Mfn2) expression by 60%—shifting the mitochondrial network toward fusion phenotypes that resist apoptotic stimuli (89). These MAM-specific adaptations are absent following single acute exercise bouts and represent a structural remodeling that requires 4–6 weeks of consistent training to manifest (90).

### 6.3 Sarcolemmal KATP channels and ischemic preconditioning

Exercise training upregulates the sarcolemmal ATP-sensitive potassium channel (sarcKATP)—specifically the Kir6.2 pore-forming subunit and the SUR2A regulatory subunit—conferring ischemic preconditioning-like protection distinct from acute exercise effects (91). After 3–4 weeks of treadmill training, rodent cardiomyocytes demonstrate 2.1-fold increased Kir6.2 protein expression and 1.8-fold increased SUR2A density, enhancing channel sensitivity to intracellular ATP/ADP ratios (92). During ischemia-reperfusion injury, hearts from trained animals exhibit accelerated sarcKATP opening, shortening action potential duration by 15%–20% and reducing calcium entry through voltage-gated channels, thereby decreasing infarct size by 45%–60% compared to sedentary controls (93).

This cardioprotective phenotype requires chronic training; acute exercise bouts do not alter Kir6.2/SUR2A protein levels (91, 94). The training effect persists for 7–10 days after cessation of exercise, suggesting epigenetic modifications including increased histone H3 acetylation at the Kcnj11 (Kir6.2) promoter (95). Functionally, the sarcKATP channel converges with the IGF-1/PI3K pathway—Akt-mediated phosphorylation at specific channel residues increases open probability during metabolic stress, representing a molecular integration of growth factor and energy-sensing signals (91, 96).

### 6.4 Metabolic, anti-inflammatory, and vascular integration

Training-induced Akt activation coordinately regulates glucose metabolism through GLUT4 translocation to the sarcolemma (increasing glucose uptake by 30%–40%) and glycogen synthase activation. Concurrently, peroxisome proliferator-activated receptor gamma coactivator 1-alpha (PGC-1*α*) expression increases 3–5-fold after 6 weeks of training, driving mitochondrial biogenesis evidenced by increased mitochondrial DNA copy number and respiratory chain complex protein expression (97). Crucially, PGC-1*α* integrates with the IGF-1/PI3K axis through direct phosphorylation by Akt, creating a feed-forward loop that maintains oxidative capacity specifically when training volumes exceed 150 min/week (98).

Training reduces myocardial nuclear factor-*κ*B (NF-*κ*B) activation by 35% and decreases TNF-α and IL-6 mRNA expression by 40%–50%, effects mediated partly through Akt-dependent inhibition of IKK*β* (9). Simultaneously, training increases myocardial superoxide dismutase 2 (SOD2) activity by 60% and catalase activity by 45%, reducing mitochondrial hydrogen peroxide production and lipid peroxidation products (4-hydroxynonenal) (99). These antioxidant adaptations require sustained PI3K signaling; pharmacological inhibition of PI3K with wortmannin during training blocks SOD2 upregulation (100).

**Figure 2:**
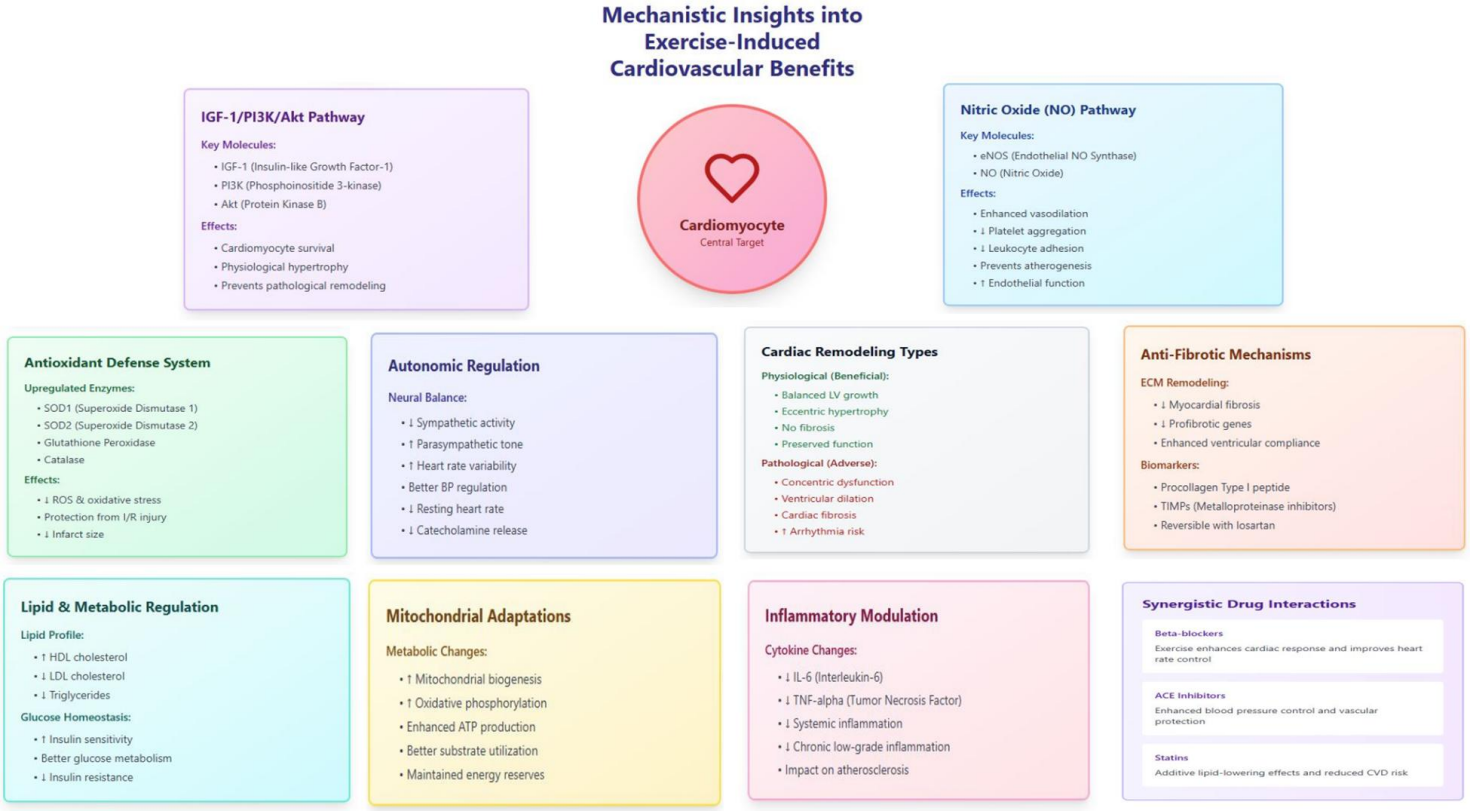
Integrated mechanisms linking exercise training to cardiovascular protection. Key pathways include (1) IGF-1/PI3K/Akt signaling, (2) eNOS/NO-mediated vascular function, (3) mitochondrial adaptations, (4) antioxidant defense, (5) autonomic modulation, (6) inflammatory regulation, and (7) anti-fibrotic remodeling, which collectively contribute to improved clinical outcomes in cardiovascular disease prevention and therapy.

In endothelial cells, training enhances Akt phosphorylation of eNOS at Ser1177, increasing nitric oxide production by 25%–35% and improving flow-mediated dilation by 2%–4%, adaptations that persist at rest unlike the transient effects of acute exercise (101). These molecular modifications collectively establish a cardioprotective phenotype distinct from untrained states (Figure 2).

## 7 Public health implications and suggestions

### 7.1 Therapeutic exercise prescription and cardiac rehabilitation

Integrating physical activity into public health policies is crucial for reducing the global impact of CVD. Based on current clinical guidelines from the WHO and the American Heart Association (AHA), adults should aim for 150–300 min of moderate-intensity aerobic exercise or 75–150 min of vigorous-intensity aerobic exercise each week, ideally paired with muscle-strengthening activities on two or more days. This proposed level of physical activity is linked to significant reductions in CVD incidence, cardiovascular mortality, and overall mortality (102). Moderate activities include brisk walking, light cycling, and dancing, while vigorous activities involve running, swimming laps, and aerobics. Significantly, even brief or sporadic sessions of physical activity, such as 10–15 min per session, can offer health advantages, making it more achievable for the general population. Tailored exercise prescriptions are critical for promoting adherence and clinical outcomes, notably for individuals with pre-existing cardiovascular conditions. Based on analyzing personal fitness levels, comorbidities, and preferences, customized exercise programs can be progressed to enhance advantages and lower risks (103, 104).

large-scale cohort studies have shown that regular participation in activities such as swimming, racquet sports, and aerobics is associated with a 36%–56% reduction in all-cause and cardiovascular mortalities compared to sedentary behavior (105). This degree of risk reduction is comparable in magnitude to that achieved with pharmacological treatments such as statins, reinforcing the role of physical activity as a cornerstone of population-level cardiovascular prevention. Adhering to current physical activity guidelines, 150–300 min of moderate-intensity or 75–150 min of vigorous-intensity activity per week, confers up to a 30% lower risk of mortality in individuals with established CAD or heart failure (7). Importantly, even sub-threshold levels of physical activity offer meaningful protection, with the most dramatic risk reductions observed when moving from a sedentary to a low-active lifestyle (75). Cardiac rehabilitation programs exemplify this approach by integrating supervised aerobic and resistance training with behavioral modifications, causing decreased risks of reinfarction and cardiovascular mortality (106, 107). Despite their reported effectiveness, these rehabilitation programs are commonly underutilized because of barriers, such as limited access, socioeconomic factors, and a reduction in referrals. Current public health studies have stressed the significance of improving physical activity at the population level via multisectoral policies that increase established environments, enhance community awareness, and decrease sedentary behaviors. For instance, longitudinal cohort literature has reported that gradually enhancing physical activity from a sedentary baseline causes the most vital decreases in cardiovascular risk, stressing that any movement is better than none (106, 108).

Public health campaigns that support active transportation, community sports, and accessible exercise facilities are essential components of approaches to prevent cardiovascular disease. Moreover, emerging evidence encourages the inclusion of HIIT as a time-efficient possibility that causes superior enhancements in cardiorespiratory fitness and endothelial function in comparison to moderate continuous exercise. However, it is critical to balance intensity and duration to avoid adverse events, especially in high-risk groups (109, 110).

The combination of clinical guidelines, study findings, and public health efforts stresses the critical significance of physical activity in both avoiding and controlling CVD. To obtain widespread cardiovascular health improvements, it is essential to determine fair access to exercise opportunities, incorporate exercise counseling into standard healthcare practices, and create environments that encourage physical activity.

### 7.2 Therapeutic exercise prescription and cardiac rehabilitation

While preventive exercise focuses on maintaining health, therapeutic exercise addresses existing cardiovascular pathology through structured, medically supervised protocols. Cardiac rehabilitation represents the gold standard therapeutic application, combining aerobic and resistance training with risk factor modification to reduce mortality by 20%–26% and hospital readmission by 30% in post-myocardial infarction (MI) patients (111).

Heart Failure with Reduced Ejection Fraction (HFrEF): The HF-ACTION trial demonstrated that supervised aerobic training (36 sessions over 3 months, progressing to home-based exercise) improves VO₂ peak by 0.6 mL/kg/min and reduces all-cause mortality/hospitalization (HR 0.89, 95% CI 0.81–0.99) when added to optimal medical therapy (22). High-intensity interval training (HIIT) at 90%–95% peak heart rate appears superior to moderate continuous training for reversing pathological remodeling in stable HFrEF, increasing left ventricular ejection fraction by 3%–5% over 12 weeks (112).

CAD: Post-MI patients beginning exercise training 1–2 weeks after revascularization show improved endothelial function and reduced residual plaque volume progression (113). Resistance training is safe 4–6 weeks post-MI, provided it begins at 30%–40% 1-RM and avoids the Valsalva maneuver (114).

Exercise-Drug Interactions: Exercise synergizes with pharmacological therapy to enhance cardiovascular protection. Physical activity improves insulin sensitivity and lipid profiles independently of statin therapy, while beta-blockers blunt maximal heart rate responses requiring adjusted prescription (targeting 50%–70% heart rate reserve rather than age-predicted maximum) (115). ACE inhibitors combined with exercise training show additive benefits in reducing fibrosis biomarkers (PICP) compared to either intervention alone (116).

Clinical Implementation: Exercise prescriptions for CVD patients require medical clearance, exercise stress testing, and ECG monitoring during initial sessions (117). The FITT-VP framework (Frequency, Intensity, Time, Type, Volume, Progression) should be individualized: initial intensity at 40%–50% VO₂ reserve progressing to 70%–85% as tolerated, with resistance training added at week 4–6 post-event (117).

## 8 Interplay between different physical activities and cardiovascular health

The interplay between different physical activities and cardiovascular health is intricate, involving a scope of exercises that offer distinct yet commonly complementary advantages (118). Based on assessing the cardiovascular impacts of various exercises, from aerobic activities, such as running and jogging, to strength-oriented exercises, such as pushups and functional movements, such as sit-to-stand transitions, individuals can gain useful insight into perfecting their heart health (119).

### 8.1 Running and jogging

Running and jogging are two of the most thoroughly assessed aerobic exercises regarding cardiovascular health. These activities raise the heart rate and promote oxygen uptake (VO₂ max), conversely, which improves cardiac output and myocardial effectiveness (32). A multitude of literature, comprising a notable cohort study conducted in 2025, has demonstrated that consistent running can decrease both all-cause and cardiovascular mortalities by improving lipid profiles, reducing blood pressure, and decreasing systemic inflammation (118). Running involves the rhythmic utilization of large muscle groups, which can cause the generation of endothelial nitric oxide, helping in vasodilation and promoting vascular health. However, it is critical to balance the intensity and volume of running, as widely high intensity in those who are not well-trained can heighten the risk of cardiac events because of temporary spikes in blood pressure and oxidative stress (120, 121).

### 8.2 Aerobic exercises: walking, brisk walking, and cycling

Participating in low-impact aerobic exercises, such as walking, brisk walking, and cycling can significantly increase cardiovascular health. Walking, even at a moderate pace, can promote heart rate variability and reduce arterial stiffness. Brisk walking provides similar advantages, such as decreasing high blood pressure and causing insulin sensitivity, notably for older adults or those with other health conditions (122, 123). It is interesting that cycling, whether outdoors or on a stationary bike, offers aerobic endurance with minimal joint strain, making it an excellent choice for individuals who cannot engage in high-impact activities. The connection between cycling and lower mortality rates, although its impact on cardiovascular mortality was less significant in comparison to swimming or racquet sports in the population literature (124, 125).

### 8.3 Strength-based activities: pushups, sit-to-stand, and resistance training

Exercises that center on strength, such as push-ups, and functional movements, such as transitioning from sitting to standing, offer cardiovascular benefits that increase aerobic workouts. These activities trigger muscle strength and endurance, which are related to promoted glucose metabolism, better vascular function, and increased cardiac autonomic balance (126). Push-ups, involving several upper-body muscles, can temporarily raise heart rate and promote the heart's ability to control oxygen demand. Sit-to-stand exercises, generally proposed for older adults to aid in encouraging independence, also have an effective influence on blood pressure management and peripheral circulation. Current meta-analyses have reported that resistance training at moderate intensities can promote lipid profiles, lower systolic blood pressure, and decrease systemic inflammation markers. Hence, integrating aerobic and resistance training is proposed to enhance cardiovascular health (32, 126, 127).

### 8.4 Recreational and team sports

Taking part in recreational and team sports, such as swimming, racquet sports, aerobics, soccer, and basketball, offers a wide scope of cardiovascular health advantages. Swimming is especially noteworthy as a low-impact exercise that engages the entire body, improves cardiorespiratory fitness, and lowers peripheral vascular resistance. Racquet sports and aerobics are linked to significant reductions in both overall and cardiovascular mortality because of their integration of aerobic and anaerobic activities. Team sports, which involve bursts of varying intensity, cause superior cardiovascular conditioning and promote autonomic function. In addition, the collaborative and competitive nature of team sports provides psychological advantages that indirectly improve heart health according to decreasing stress and depression (128, 129).

### 8.5 Functional movements: sit and stand

Functional movements performed daily, such as sit-to-stand exercises, are increasingly recognized for their influence on cardiovascular health, notably among older adults. These exercises increase lower-body strength, balance, and endurance, which promotes circulation and cardiovascular resilience. Although studies are still appearing, the literature reveals that enhancing the frequency and speed of these movements can promote heart rate recovery and decrease arterial stiffness, stressing the potential of simple daily activities to encourage cardiovascular function (130, 131).

Collectively, the scope of physical activities discussed offers synergistic cardiovascular advantages. Aerobic exercises increase cardiopulmonary effectiveness and endothelial function, strength-based activities improve metabolic and vascular health, and functional movements promote overall cardiovascular resilience and quality of life (132, 133). Public health guidelines are increasingly suggesting an integration of these activities to enhance cardiovascular protection. It is worth noting that new literature evidence also stresses the significance of individualized exercise plans that consider individual fitness levels, health conditions, and personal preferences to facilitate adherence and outcomes (134).

Earlier studies have revealed that a varied exercise regimen, comprising running, jogging, walking, cycling, strength training, recreational sports, and functional activities, offers an integrative method to cardiovascular health. This difference not only increases physiological advantages, but also facilitates psychological well-being, being advantageous for a sustainable and heart-healthy lifestyle (82).

## 9 Relationship with gym workouts

The correlation between gym workouts, such as resistance training and powerlifting, and cardiovascular health is intricate and multifaceted. Growing literature stresses the vital role these physical activities play in both avoiding and controlling CVD (135, 136). Although aerobic exercise has traditionally been stressed by its cardiovascular advantages, current scientific findings advocate for the inclusion of resistance training and powerlifting as vital elements of an integrative cardiovascular health approach (137, 138).

### 9.1 Gym workouts and cardiovascular health

Gym sessions commonly consist of a blend of aerobic exercises, strength training, and flexibility routines. This combined approach yields combined benefits for cardiovascular well-being. Consistent gym participation, which includes the utilization of cardiovascular equipment, such as treadmills, elliptical trainers, and stationary bikes, improves cardiorespiratory fitness (CRF), causes maximal oxygen uptake (VO₂ max), and improves heart function (139, 140). Moreover, engaging with resistance machines and free weights helps in establishing muscular strength, which indirectly supports cardiovascular health according to impacting the metabolic and inflammatory pathways related to heart disease. A systematic review conducted in 2025 found that gym-based integrated workouts can decrease resting blood pressure, promote lipid profiles, and increase insulin sensitivity, providing significant advantages for middle-aged and older adults with increased cardiovascular risk (141, 142).

### 9.2 Resistance training and cardiovascular benefits

Resistance training (RT) can involve exercises that center on improving muscle strength and endurance via repeated muscle contractions against external resistance. The cardiovascular benefits of RT are obtaining recognition, including enhancements in blood pressure, glucose metabolism, lipid profiles, and body composition. Unlike aerobic exercise, which mainly causes volume overload in the heart, RT typically causes concentric cardiac remodeling, defined according to an enhancement in left ventricular wall thickness without chamber dilation (143). This type of remodeling improves myocardial contractility and stroke volume while maintaining diastolic function. Recent meta-analyses show that participating in RT at moderate intensity (two to three sessions per week) is linked to a 10%–20% decrease in cardiovascular mortality and the incidence of CVD events, independent of aerobic exercise (13).

Notably, resistance training improves vascular endothelial function by increasing nitric oxide-induced vasodilation and decreasing arterial stiffness, causing better blood pressure modulation. The effect of RT on systemic inflammation is also significant, as it decreases pro-inflammatory cytokines, such as IL-6 and TNF-alpha, which are critical in the progression of atherosclerosis (144). Based on another study published in 2025, resistance training not only improves physical function but also effectively impacts sleep and mood, factors that are acknowledged to impact cardiovascular outcomes (141).

### 9.3 Powerlifting and cardiovascular considerations

Powerlifting, characterized by maximal or near-maximal loads for low repetitions (1–5 reps), imposes unique cardiovascular demands distinct from general resistance training. The Valsalva maneuver, commonly employed during heavy lifts, causes acute increases in systolic blood pressure (up to 300–400 mmHg) and heart rate reduction via baroreceptor reflex, followed by post-lift hypotension (145). Chronic powerlifting training induces concentric left ventricular remodeling (increased wall thickness without chamber dilation), which differs from the eccentric remodeling seen in endurance athletes (146).

While moderate powerlifting improves vascular endothelial function and reduces resting blood pressure long-term, extreme loads without aerobic conditioning may increase arterial stiffness (147). Screening for pre-existing cardiovascular conditions (e.g., aortic root dilation, Marfan syndrome) is essential, as the acute hemodynamic stress of competition lifts poses risks for susceptible individuals (148). Powerlifters should integrate aerobic conditioning (150 min/week moderate intensity) to mitigate potential detrimental effects on arterial compliance and cardiac autonomic balance (149).

### 9.4 Synergistic impacts of combined training

The most significant cardiovascular advantages must be derived from training programs that incorporate both aerobic and resistance exercises. These integrated regimes not only enhance cardiac output and peripheral vascular function but also enhance metabolic profiles (150, 151). Evidence from clinical guidelines and randomized controlled trials displays that such programs can decrease cardiovascular mortality, promote quality of life, and decrease hospital admissions in patients with established CVD (152, 153). The constructive crosstalk between these exercises is due to aerobic training's ability to facilitate oxygen delivery and endothelial health. In contrast, resistance training helps control components of metabolic syndrome and promotes musculoskeletal integrity, which is vital in sustaining functional independence (154, 155). To combine resistance training and powerlifting into gym routines that encourage cardiovascular health, it is essential to customize the approach according to an individual's fitness level, health condition, and risk factors (137, 156). For most adults, engaging in resistance exercises targeting all major muscle groups at least twice a week, in conjunction with moderate aerobic activities, is in line with current physical activity suggestions. Powerlifting can be performed safely with proper supervision, gradual progression, and concurrent aerobic conditioning (157). Health professionals and exercise specialists assess cardiovascular risk, monitor blood pressure responses, and instruct individuals on proper techniques to enhance advantages and decrease risks (158, 159).

## 10 Conclusion

The concept of “exercise mimetics” pharmacological compounds that activate molecular pathways downstream of exercise without physical activity represents an emerging frontier for patients unable to engage in regular training. 5-aminoimidazole-4-carboxamide ribonucleotide (AICAR), an AMPK activator, mimics endurance training by inducing mitochondrial biogenesis and enhancing glucose uptake through PGC-1*α* activation, though with modest effects compared to voluntary wheel running in rodent models. GW501516 (PPAR*δ* agonist) increases oxidative capacity and fatty acid oxidation in skeletal muscle, producing endurance-like adaptations without exercise training. More clinically relevant, SGLT2 inhibitors and metformin partially replicate exercise-induced metabolic shifts by activating AMPK and promoting ketone body metabolism, offering cardioprotective benefits in heart failure patients. However, current mimetics cannot replicate the integrated hemodynamic, neural, and structural cardiac adaptations of physical activity. Future development requires multi-target approaches combining AMPK, PPAR*δ*, and PI3K pathway activators to approximate the polypharmacology of exercise.

Advances in multi-omics technologies enable mechanistic dissection of exercise responses at systems biology levels. Genomics identifies single-nucleotide polymorphisms (e.g.,ACE I/D, ACTN3 R577X) that modulate training responsiveness, explaining 30%–50% of inter-individual variance in VO₂ max adaptations. Transcriptomics reveals circulating microRNAs (miR-1, miR-133a, miR-206) as biomarkers of myocardial adaptation, with exercise-trained athletes showing distinct miRNA signatures predictive of physiological hypertrophy. Metabolomics identifies exercise-induced metabolites [e.g., *β*-aminoisobutyric acid (BAIBA), kynurenine] mediating cross-tissue communication between muscle, adipose tissue, and heart. Integration of these datasets through machine learning algorithms promises precision exercise prescriptions tailored to individual genetic and metabolic profiles, optimizing cardiovascular outcomes while minimizing risks such as atrial fibrillation in susceptible genotypes.
